# Burnout and associated factors among nurses in psychiatric and general tertiary hospitals in Botswana: A cross-sectional study

**DOI:** 10.1177/20503121241272636

**Published:** 2024-10-09

**Authors:** Keneilwe Molebatsi, Otsetswe Musindo, Kagiso Bojosi, Nduku Wambua, Anthony A Olashore

**Affiliations:** 1 Faculty of Medicine, Department of Psychiatry, University of Botswana, Gaborone, Botswana; 2 Department of Psychiatry, Nelson Mandela School of Clinical Medicine, University of Kwa-Zulu Natal, Durban, South Africa; 3 Selibe Phikwe Government Hospital, Ministry of Health and Wellness, Selibe Phikwe, Botswana; 4 Sbrana Psychiatric Hospital, Lobatse, Botswana; 5 Department of Clinical, Neuro, and Developmental Psychology, Amsterdam Public Health Research Institute, Vrije Universiteit Amsterdam, Amsterdam, The Netherlands

**Keywords:** Burnout, nurses, job satisfaction, personality, Botswana

## Abstract

**Introduction:**

Research has consistently documented that nurses are at a higher risk for developing burnout syndrome due to the nature of their jobs. The high prevalence of burnout among nursing staff negatively affects healthcare delivery. Nurses experiencing burnout cannot provide quality care or actively participate in health promotion, and those experiencing emotional exhaustion are unlikely to be able to tend to the emotional needs of patients.

**Objective:**

The study aimed to determine the prevalence and factors associated with burnout syndrome among nursing staff in Botswana referral general and psychiatric hospitals.

**Methods:**

A cross-sectional survey was conducted among 249 nursing staff working in a referral psychiatric and general hospital. The job satisfaction survey, Maslach burnout inventory, and the OCEAN-20 were used to assess job satisfaction, burnout occurrence, and personality traits, respectively. A researcher-designed sociodemographic questionnaire was used to capture demographic data. Variables identified to be associated with burnout on bivariate analyses were entered into a regression analysis to determine to what extent these factors predicted burnout.

**Results:**

The prevalence of emotional exhaustion, depersonalization, and reduced personal accomplishment was 65.7%, 56.9%, and 54%, respectively. Being in a general hospital, neuroticism, poor operating condition, and poor communication predicted emotional exhaustion, *R*^2^ = 28%, *F* (9, 229) = 9.937, *p* < 0.001. Depersonalization was predicted by neuroticism and poor operating conditions, *R*^2^ = 17%, *F* (9, 229) = 4.709, *p* < 0.001. Reduced personal achievement was significantly predicted by the number of people in the household, time at the current hospital, agreeableness, and nature of work, *R*^2^ = 21%, *F* (7, 203) = 7.875, *p* < 0.001.

**Conclusions:**

Our findings highlight the need for interventions to be developed to address burnout among nursing staff to optimize healthcare delivery.

## Introduction

The most accepted definition of burnout syndrome is based on the socio-psychological perspective of Maslach and Jackson.^1^ In 2019, the World Health Organization publicly stated that burnout will be included in the International Classification of Diseases (ICD-11), which guides medical providers in diagnosing diseases. However, it is not classified as a medical condition but an occupational health-related phenomenon. This considers burnout as a reaction to chronic emotional tension caused by over-dealing with people.

Burnout is a construct formed by three related but independent dimensions: emotional exhaustion, depersonalization, and diminished personal accomplishment.^2^ Depersonalization is the development of distrustful and pessimistic attitudes toward colleagues and clients. Diminished personal accomplishment results in individuals believing they are ineffective in their jobs, leading to reduced performance,^3^ whereas emotional exhaustion is characterized by feeling emotionally and/or physically depleted because of continued stress and demands from an individual’s occupation. Complications of burnout include affective problems, multiple psychosomatic complaints, attitude and behavioral problems such as hostility, irritability, and isolation.^4,5^

The healthcare industry has been widely acknowledged as stressful because of human resource shortages and high demands. Previous research has consistently reported that nurses are at high risk for developing burnout syndrome compared to other health care professionals due to the nature of their jobs.^6^ Comparatively, nurses are the first line of contact; they spend the most time with patients and are constantly exposed to the emotional strains of dealing with the sick and dying. Such stressors, when left unchecked, lead to burnout.^7^

Several studies conducted in Africa have reported high rates of burnout among healthcare workers, particularly nurses. Nurses in a Nigerian general hospital reported high levels of burnout, with 39.1%, 29.2%, and 40% reporting emotional exhaustion, depersonalization, and reduced personal accomplishment, respectively.^8^ Similarly, high levels of emotional exhaustion and depersonalization were reported among nurses in three East African countries (Kenya, Tanzania, and Uganda).^9^ In Southern Africa, Researchers in Malawi found significantly high numbers of nursing staff with burnout across all domains in Maslach’s Burnout Inventory (MBI).^10^ In Zimbabwe, 39% of intensive care unit (ICU) nurses were found to have severe feelings of emotional exhaustion.^11^ Comparatively, 45.8% of nursing staff in a referral hospital in South Africa reported high levels of burnout on the emotional exhaustion subscale of the MBI.^12^ We only found one study in Botswana that investigated associations between healthcare worker participation in workplace wellness activities and job satisfaction, occupational stress, and burnout among staff employed in public health facilities; the study participants included HCWs not providing direct human services.^13^

The high prevalence of burnout among nursing staff affects healthcare delivery. Nurses experiencing burnout cannot provide quality care or actively participate in health promotion. Nurses experiencing emotional exhaustion are unlikely to be able to tend to the emotional needs of patients^14^ and may experience mental health complications such as suicidal ideation^15^ Although Africa accounts for 25% of the world’s burden of disease, it only constitutes 3% of the world’s health care personnel.^16^ Therefore, healthcare workers in resource-limited countries such as Botswana have a high risk of developing burnout due to inadequate facilities or staffing, leading to a huge work overload. Burnout poses significant consequences on social and occupational functioning and, most importantly, low productivity in health care delivery. Determining the prevalence and risk factors of burnout among nursing staff is important in laying a good framework for health services planning and improved service delivery.

Experience of burnout is determined by both personal variables and organizational variables.^17^ Job satisfaction has been reported as one of the important predictors of burnout among nursing staff; a study among Bangladeshi nurses revealed that those with delayed payments and limited resources were found not to be satisfied with their jobs, with consequent high rates of burnout.^18^ Sociodemographic factors such as younger age,^19,20^ being female,^21^ and being married have been reported as risk factors for burnout. Neuroticism and organizational variables such as high workload and emotional demands at work, for example, dealing with the mentally unwell, also increase the risk of burnout.^17^ Prevalence and risk factors of burnout may, therefore, differ according to the type of facility where one is based and varied job prescriptions. Psychiatric nursing is peculiar because the nurses have to care for the mental health care users and their caregivers’ emotional demands.^22,23^ Moreover, they have to sometimes deal with difficult behaviors, for example, uncooperative, violent, or suicidal patients, which is not the case with general medical nursing.^24–26^ Additionally, burnout rates among psychiatric nurses may be higher due to the duration of contact psychiatric nurses have with their patients compared to their counterparts.^22,24,27^ The aim of this study was to determine the prevalence and associated risk factors of burnout syndrome among nursing staff in a referral general and psychiatric hospital in Botswana.

## Methods

### Study design and site

The study utilized a cross-sectional descriptive design and was conducted at two tertiary facilities in Botswana. Sbrana Psychiatric Hospital is the only referral psychiatric hospital in Botswana. The hospital also serves as a training center for medical and nursing students. Sbrana Psychiatric Hospital has a capacity of 300 beds with ten wards (4 female and 6 male wards). Princess Marina Hospital started operating in 1967 and is Botswana’s largest referral general hospital. It has a bed capacity of more than 500 beds. The hospital has several specialized departments, such as obstetrics and gynecology, pediatrics, surgery, orthopedics, and ophthalmology. Nursing staff rotate annually between the wards but may stay for more than a year in one ward.

### Study population

The total sample size of 235 was determined using epi-info software at 95% CI, 50% expected frequency, and an estimated finite population of 600 nurses in both facilities. The study targeted nursing staff employed at PMH and SPH during the data collection period; May to December 2019. To be included in the study, participants had to give informed consent (verbal or written) and should have worked at either facility for a period of 6 months or more. The researchers used convenience sampling to recruit participants, approaching nurses who were on day duty during data collection. Because shifting occurs weekly, we expected to get a representative sample of nurses working day or night shifts.

### Measures

*Researcher-designed sociodemographic questionnaires* were used to collect data on variables such as age, gender, educational level, employment status, monthly income, and marital status.

*Maslach burnout inventory (MBI)—*Burnout syndrome was measured using the Maslach Burnout Inventory.^1^ It comprises 22 items with a seven-point Likert response scale from zero (“Never”) to six (“every day”). The MBI has three dimensions: emotional exhaustion (EE; nine items), depersonalization (D; five items), and personal accomplishment (PA; eight items) and has demonstrated acceptable validity and reliability for South African nurses,^28^ which is a similar population to the one being studied.

*Job satisfaction survey (JSS)—*The job satisfaction survey was used to measure nursing staff job satisfaction. The tool was developed as a measure of employee job satisfaction applicable specifically to human service, public, and nonprofit sector organizations;^29^ it is therefore suitable for health service personnel working in government-run facilities. Each item on the interview is a statement that the employee is asked to endorse using a 6-point scale ranging from 0 (disagree very much) to 6 (agree very much). The tool measures nine dimensions of job satisfaction: nature of work, quality of supervision, promotion, communication, relationship with coworkers, fringe benefits, contingent rewards, operating conditions, and pay. The JSS total score categorizes employees into three groups: dissatisfied (36–108), ambivalent (109–144), and satisfied (145–216). The JSS has demonstrated high internal consistency and a high test–retest reliability (0.71).^30^ The JSS was not validated in our setting; however, we calculated the Cronbach’s alpha, which was 0.79 for the JSS.

*OCEAN.20 assessment*—The OCEAN.20 is a 20-item Five-Factor Personality Measure.^31^ The OCEAN.20 assesses each of the Big Five personality factors with four items, and participants endorsed items on an ordered agreement scale from “1: Extremely uncharacteristic of me” to “7: Extremely characteristic of me.” The OCEAN has demonstrated very good reliability on all five factors: Openness (α = 0.78), Conscientiousness (α = 0.88), Extraversion (α = 0.87), Agreeableness (α = 0.81), and Neuroticism (α = 0.77).^31^ Although the OCEAN was not validated in our setting, we found that certain factors demonstrated good reliability in our sample: Openness (α = 0.646), Conscientiousness (α = 0.797), Extraversion (α = 0.643), Agreeableness (α = 0.81), and Neuroticism (α = 0.741).

### Data collection procedures

The study was conducted with the permission of the University of Botswana Institutional Review Board (UBIRB) (UBR/RES/IRB/BIO/086), the Ministry of Health (HPDME: 13/18/1), and the management and ethics committees of Sbrana Psychiatric Hospital (SPH 4/2/12 I) and Princess Marina Hospital. In addition, all participants gave written informed consent. Before the commencement of data collection, KM met with the head of facilities and units to introduce the study. Trained research assistants (RA) then went to each department and explained the study further. Staff willing to participate were given a consent document form to read about the study further and gave either verbal or written consent. Data were collected according to participants’ preference; some preferred the RA to administer the tools, while others preferred to be left with the questionnaires and submit them upon completion. Data were collected using a researcher-designed sociodemographic questionnaire, the abbreviated MBI, JSS, and the OCEAN.20. For those who preferred the RA-administered interviews, data collection was done in a private meeting room, which is in hospital wards at the participant’s convenience time. Nursing staff on night shifts were interviewed during day duties.

### Data analysis

All statistics were performed with SPSS 27.0 software (SPSS Inc., Chicago IL, USA). While we checked for the normality of the outcome variables, we used parametric statistics because of our large sample size (greater than 50).^32^

Although our histogram doesn’t display a perfect bell-shaped curve, it is reasonably normal with a few outliers, as seen in our Q-Q plot. The plot showed no real clustering of points, with most of them centered around the zero line. To ensure that these outliers don’t unduly influence our model results, we referred to Tabachnick and Fidell’s^33^ recommendation and checked the values for Cook’s Distance. It suggests that cases with values larger than 1 are significant deviations and could be a problem. However, in our case, all the values were less than 1. Specifically, for personal achievement, it was 0.14; for depersonalization, it was 0.079; and for emotional exhaustion, it was 0.20.

For the descriptive statistics, the continuous sociodemographics, such as age and other clinical variables, including burnout and personality scores, were presented with means and standard deviations, while the categorical variables (e.g., gender) were reported as percentages. For further analysis, some categorical variables, such as marital status, were recategorized as single and married. Exploratory analysis was conducted using independent samples *t*-tests to investigate the relationships between categorical variables, such as gender and marital status, and outcome variables, which are the subscales of burnout, namely emotional exhaustion, depersonalization, and personal achievement.

A Pearson’s correlation was run to assess the relationship between the burnout subscales, perceived job satisfaction, personality, and demographic variables. Variables found to correlate with burnout on bivariate analysis at a *p*-value of less than 0.05 were included in the regression model. In this study, we utilized a one-step multipleregression analyses to predict the burnout subscales from an array of factors, including job satisfaction, personality, and demographic information. For each of the three burnout subscales, we conducted individual models. To avoid model overfitting, we employed the formula *N* > 50 + 8 *m* (where *m* = number of independent variables) as recommended by Tabachnick and Fidell.^33^ This criterion stipulates that a minimum of 90 cases is necessary for five independent variables. Furthermore, we ensured that multicollinearity was addressed by maintaining a tolerance value of less than 0.10 and a VIF value of above 10. All associations with a *p*-value of less than 0.05 were deemed statistically significant.^33^

## Results

### Participant characteristics

We recruited 249 nurses, 41% of whom worked at the mental health facility. The mean (SD) age of our participants was 34.57 (9.18), and women made up 64.3% of them. Table 1 describes our participants.

**Table 1.** Participant demographics.

Variables		*N* (%)
Sex	Female	160 (64.3)
	Male	88 (35.3)
Hospital	Mental health	102 (41)
	General	147 (59)
Marital status	Single	103 (41.5)
	Married	99 (39.9)
	Divorced	3 (1.2)
	Widowed	3 (1.2)
	Cohabiting	3 (1.2)
	Others	31 (12.5)
Level of education	Diploma	172 (69.1)
	Bachelors	55 (22.1)
	Graduate	18 (7.2)
Current designation	Junior	63 (25.3)
	Middle	158 (63.5)
	Senior	4 (1.6)
	*N*	Mean (SD)
Age	242	34.57 (9.2)
No of children	236	1.48 (1.3)
No. of people in household	238	2.68 (1.8)
Time at current hospital	248	2.21 (0.9)

*t*: independent *t*-test; MD: mean difference; SD: standard deviation.

### Prevalence of burnout and job satisfaction

This study found the prevalence of burnout to be 65.7%, 56.9%, and 54% in emotional exhaustion, depersonalization, and reduced personal accomplishment, respectively.

Only 19.7% (49) nurses were satisfied with their jobs, with 9.6% (24) claiming to be satisfied with their pay, 21.3% (53) with their promotion, 14.9% (37) with fringe benefits, 46% (116) with contingency reports, and 59.4 (148) with operating conditions.

Independent samples *t*-tests were run to determine whether there were differences in burnout and job satisfaction scores between the hospitals and sex. There were differences in burnout scores between hospitals; nurses in the general hospital experienced more burnout than those in the psychiatric hospital. The mean difference between genders was not statistically significant. See Table 2.

**Table 2.** Total burnout, job satisfaction, and their relationships with hospital and sex.

Variables	Independent variables	*N*	Mean	Std. deviation	*t*	*p*-Value
Burnout total	Hospital					
	Sbrana	98	61.4	17.3	−3.78	**<0.01**
	Marina	147	70.8	20.3		
Total job satisfaction	Sbrana	99	119.7	25.8	−4.88	**<0.01**
	Marina	147	133.4	13.0		
Burnout	Sex					
	Male	88	65.6	20.8	−0.96	0.336
	Female	159	68.1	19.1		
Total job satisfaction	Male	88	128.4	19.8	0.32	0.752
	Female	160	127.6	20.6		

Significant *p*-value in Bold.

t: independent t-test; MD: mean difference; SD: standard deviation.

### Factors associated with burnout

The bivariate analysis in Supplemental Table 1 reveals that the current position, specifically junior staff, was associated with the reduced personal achievement subscale of the MBI (*t* = 2.13; *p* = 0.036). Working in Princess Marina Hospital was associated with emotional exhaustion (*t* = −3.934, *p* < 0.01) and depersonalization (*t* = −1.93, *p* = 0.045). Likewise, neuroticism (*r* = 0.30, *p* = 0.01), poor operating condition (*r* = 0.36, *p* = 0.01), and poor communication (*r* = 26, *p* = 0.01) were observed to have a moderately strong association with the emotional exhaustion subscale of burnout in the Supplemental Table 2. Other results of the bivariate analyses are shown in Supplemental Tables 1 and 2. Factors associated with the subscales of burnout on bivariate analysis, *t*-test, and Pearson’s correlation were included in the model, as shown in Table 3.

**Table 3.** Linear regression showing the relationship between the outcome and independent variables.

Variables		*B*	95% CI for B		*p*-Value	*R* ^2^	*F*	Intercept	Collinearity statistics	
Outcomes	Independent variables		Lower	Upper					Tolerance	VIF
Emotional exhaustion	Hospital (Marina)	0.16	1.26	8.12	0.**007**	0.28	9.93	−19.5	0.87	1.15
	Openness	0.05	0.21	0.46	0.468				0.77	1.29
	Conscientiousness	0.07	0.17	0.62	0.272				0.88	1.13
	Neuroticism	0.23	0.28	0.87	**<0.001**				0.89	1.13
	Pay	0.01	0.48	0.51	0.845				0.83	1.19
	Supervision	−0.08	0.89	0.24	0.252				0.74	1.34
	Contingency reward	0.01	0.37	0.45	0.853				0.79	1.27
	Operating condition	0.31	0.61	1.46	**<0.001**				0.75	1.33
	Communication	0.14	0.04	0 .96	**0.032**				0.79	1.26
Depersonalization	Hospital (Marina)	0.05	−0.95	1.97	0.492	0.16	4.71	−6.109	0.86	1.16
	Openness	0.07	−0.07	0.21	0.323				0.84	1.19
	Neuroticism	0.19	0.07	0.31	**0.003**				0.89	1.13
	Pay	0.04	−0.13	0.26	0.519				0.83	1.20
	Supervision	0.05	−0.16	0.33	0.477				0.72	1.39
	Contingency rewards	−0.01	−0.19	0.17	0.900				0.77	1.29
	Operating conditions	0.17	0.03	0.40	**0.024**				0.70	1.42
	Communication	0.12	−0.02	0.37	0.077				0.79	1.26
	Co-worker	0.01	−0.22	0.26	0.894				0.70	1.43
Personal achievement	No. of people in household	−0.17	−1.26	−0.16	**0.012**	0.21	7.88	19.4	0.92	1.09
	Time at current hospital	−0.17	2.61	0.20	**0.023**				0.71	1.42
	Openness	0.07	−0.10	0.30	0.306				0.83	1.21
	Conscientiousness	0.01	−0.30	0.33	0.923				0.48	2.07
	Agreeableness	0.26	0.18	0.86	**0.003**				0.52	1.91
	Nature of work	0.17	0.08	0.62	**0.012**				0.93	1.08
	Current position	−0.01	−2.74	2.31	0.869				0.68	1.47

*B*: unstandardized regression coefficient; CI: confidence interval; *R*^2^: coefficient of determination; VIF: Variance Inflation Factor.

* Significant *p*-value in Bold.

After regression analysis, we found that hospital (working in Princess Marina Hospital), neuroticism, poor operating condition, and poor communication statistically significantly (positively) predicted emotional exhaustion, with total variance explained by the model being 28%, *F* (9, 229) = 9.937, *p* < 0.001. Depersonalization was significantly predicted by neuroticism and poor operating conditions; the total variance explained by the model was 17%, *F* (9, 229) = 4.709, *p* < 0.001. Personal achievement was negatively predicted by the number of people in the household and time at the current hospital and positively predicted by agreeableness and nature of work; the total variance explained by the model being 21%, *F* (7, 203) = 7.875, *p* < 0.001. Regression coefficients and other statistics can be found in Table 3.

## Discussion

The aim of this study was to determine the prevalence of burnout and associated risk factors among nursing staff in a referral general and psychiatric hospital in Botswana. Nursing professionals are more vulnerable to burnout compared to other professionals since their work requires constant emotional contact with other human beings.^6^ The results of this study indicate a high level of burnout syndrome among nurses in the hospitals that participated. MBI scores indicated that 65.7%, 56.9%, and 54% of the respondents experienced high burnout in emotional exhaustion, depersonalization, and reduced personal accomplishment, respectively. When comparing those in the high category alone, the prevalence among our participants was higher than what has been reported in previous studies in Southern Africa.^11,28,34^ The variance could be explained by the different study settings and differences in demographic variables, which have been shown to be associated with varying degrees of burnout.^6,35^ Our findings were, however, comparable to a study conducted by Engelbrecht et al.^36^ in South Africa, where 98.1% of the respondents scored high on the assessment of depersonalization. Drafke Kossen and Sahraian^37^ explain that the work environment of nurses is a significant contributor to burnout. Our findings showed slight mean differences among the average scores recorded for each hospital of burnout syndrome among nurses. Differences in the work environment between a general referral and a psychiatric hospital may account for the difference.

Specific to our study setting, there are severely limited human resources, resulting in underutilization of the available wards, overcrowding of the open wards, and, therefore, an increased workload. Botswana’s resource-limited setting also contributes to the high prevalence of burnout. Nursing staff must be innovative with the little available to provide the best care.

We did not find any association between sociodemographic factors and burnout scales. Age did not reveal a significant association with the burnout scales. In contrast, burnout has been found to be higher among female nurses, unmarried nurses, younger ages less than 35, and those who are in the rank of senior nurses.^38^ Our results did not agree with Maslach’s report that the prevalence of burnout is more common in the younger age groups.^38^ This may be explained by the fact that the mean age of our participants was 34.57 (9.18); many nurses have already gained experience in the profession at this age. Nurses who have less experience are more likely to experience burnout. This is in congruence with previous studies that confirm that older nurses with more years of working experience are less likely to experience burnout than younger nurses.^39–41^

Most nurses in this study reported moderate job satisfaction, which confirms previous results concerning nurses.^42^ Minority of nurses reported satisfaction with their pay (9.6%), promotion (21.3%), fringe benefits (14.9%), and contingency reports (46%), while 59.4% reported satisfaction with operating conditions. There is a clear indication of a need to understand the factors involved in job satisfaction to improve nurses’ well-being. A systematic review by Lu et al.^43^ found degree of cohesion, perception of staff organization, salaries, opportunities for advancement, extrinsic reward, autonomy, communication, recognition, working conditions, professional practice, organizational support and practices, physical and psychological responses to work, as well as patient relationships to lead to high job satisfaction. Therefore, it is important to note that a nurse who is dissatisfied with their work may lose interest and commit less, leading to poor quality of care.

Poor operating conditions and poor communication at the place of work predicted emotional exhaustion consistent with previous findings. Communication barriers may precipitate frustrations, leading to emotional exhaustion. Bahrain and colleagues examined the effects of poor communication on employees and organizations and concluded that poor communication may result in employees feeling unheard or devalued, with resultant disengagement and missed opportunities for growth and innovation.^44^ Another study to assess the effects of communication skills training on burnout conducted among nurses in Iran found that the intervention group had less burnout after the intervention compared to those in the control group. This study suggested that communication skills training could be used to reduce burnout among nurses.^45^

Poor workplace operating conditions also predicted depersonalization. Nursing staff in Botswana have no influence on where they are placed. For example, a psychiatric nurse may be posted in an orthopedic ward. Allowing staff to be actively involved in crafting their work has been found to be beneficial in preventing burnout^46^; it is therefore not surprising that operating conditions predicted depersonalization in our study.

Our study confirmed the role of personality traits in influencing the experience of burnout. Strong associations were found between different personality traits and all three dimensions of burnout. We found that neuroticism predicted emotional exhaustion, depersonalization, and reduced personal achievement, which was similar to a study carried out by Ang et al.^47^ and Cañadas-De la Fuente et al.^17^ Neuroticism is a factor with high vulnerability potential that gives rise to negative emotions, maladjustment, and increased individual sensitivity to stress^48^ accelerating the burnout process through a disproportionally pronounced feeling of stress.^49^ Personal achievement was positively correlated with agreeableness. The trait of agreeableness has been linked with the development of socially oriented tendencies, which in turn lead to enhanced teamwork, improved job performance, and increased productivity.^50^ Research has demonstrated that improving one’s agreeableness can significantly impact various aspects of personal and professional development, including career growth, overall well-being, and personal growth.^51^ Nurses with agreeableness are flexible, sympathetic, more cooperative, and trusting.^17^

There was a variance in burnout experience between the two hospitals, with nurses in the medical hospital reporting higher rates of burnout than nurses in the psychiatric hospital. These could be explained by the difference in professionals who work at the two facilities. The environment and type of disorders managed at the referral general hospital dictate constant monitoring of patients; patients being seen at the tertiary hospital present with severe conditions; consequently, the nurses are exposed to higher mortality rates than those at a psychiatric hospital. Moreover, the cost of living in the capital city where the general hospital is situated is considerably higher than the one in Lobatse, but nurses in both facilities earn the same salary; this is further evidenced by the low satisfaction rate about pay among the nurses at the general hospital.

### Limitations

This study has limitations that should be considered when interpreting the results. The cross-sectional design adopted only allows for association and not causal relationships. Nurses were recruited from referral hospitals located in the capital city of Gaborone, one acute care hospital, and hence, results might not be generalizable to other settings such as rural, community, and primary care settings.

There are other potential sources of biases that suggest the need to cautiously interpret the findings of this study, and these include the following:

The use of a convenient sample selection method may introduce selection bias, potentially leading to a lack of fairness by excluding individuals who could have been selected under random conditions. Furthermore, the timing of the selection process, particularly when some individuals were on leave, could have resulted in the omission of individuals with the relevant outcome of interest.

The study is susceptible to recall bias due to the reliance on participant questionnaires, which necessitate the recollection of past symptoms or events. This susceptibility is particularly relevant for individuals exhibiting depressive symptoms.

We did not conduct a full validation of the instruments; however, while most of them have been used in settings similar to ours, we calculated the Cronbach’s alpha for most of them.

Also, given the utilization of self-report tools, the potential for bias in symptom reporting must be considered. This method may have resulted in either over- or under-reporting of symptoms, particularly in individuals with co-occurring psychiatric symptoms, such as behavioral manifestations that were not assessed.

## Conclusion

This study sheds light on the phenomenon of burnout among nurses in Botswana, contributing to a better understanding of its prevalence and underlying factors. Specifically, our findings highlight the significant role of personality traits in shaping the experience of burnout in this context. The findings of our study highlight the urgent need for targeted interventions and preventive measures to mitigate burnout among healthcare professionals in Botswana. As one of the few studies to have explored the subject, our research emphasizes the gravity of the issue and the importance of addressing it as a critical public health concern. To this end, we recommend the implementation of targeted training programs, mental health support services, and other initiatives aimed at enhancing healthcare workers’ well-being and job satisfaction. These may include improved remuneration packages, better work environments, and increased staffing. Also, it is important to establish policies that ensure the proper placement of staff based on their qualifications and specializations. For example, in Sbrana Psychiatric Hospital, it was observed that a significant number of nurses were midwives and general nurses. This may have had an adverse impact on their job satisfaction despite satisfactory remuneration. Furthermore, it is imperative that organizational policies prioritize the detection and management of burnout while also investing in human resources, as burnout can lead to reduced productivity, increased absenteeism, and a decline in overall work performance. Our study provides valuable insights into the challenges faced by healthcare professionals in Botswana and underscores the significance of addressing burnout to sustain a healthy and productive workforce.

## Supplemental Material

sj-docx-1-smo-10.1177_20503121241272636 – Supplemental material for Burnout and associated factors among nurses in psychiatric and general tertiary hospitals in Botswana: A cross-sectional study Supplemental material, sj-docx-1-smo-10.1177_20503121241272636 for Burnout and associated factors among nurses in psychiatric and general tertiary hospitals in Botswana: A cross-sectional study by Keneilwe Molebatsi, Otsetswe Musindo, Kagiso Bojosi, Nduku Wambua and Anthony A Olashore in SAGE Open Medicine

sj-docx-2-smo-10.1177_20503121241272636 – Supplemental material for Burnout and associated factors among nurses in psychiatric and general tertiary hospitals in Botswana: A cross-sectional study Supplemental material, sj-docx-2-smo-10.1177_20503121241272636 for Burnout and associated factors among nurses in psychiatric and general tertiary hospitals in Botswana: A cross-sectional study by Keneilwe Molebatsi, Otsetswe Musindo, Kagiso Bojosi, Nduku Wambua and Anthony A Olashore in SAGE Open Medicine

sj-docx-3-smo-10.1177_20503121241272636 – Supplemental material for Burnout and associated factors among nurses in psychiatric and general tertiary hospitals in Botswana: A cross-sectional study Supplemental material, sj-docx-3-smo-10.1177_20503121241272636 for Burnout and associated factors among nurses in psychiatric and general tertiary hospitals in Botswana: A cross-sectional study by Keneilwe Molebatsi, Otsetswe Musindo, Kagiso Bojosi, Nduku Wambua and Anthony A Olashore in SAGE Open Medicine

## References

[1] Maslach C Jackson SE. The measurement of experienced burnout. J Organ Behav 1981; 2: 99–113.

[2] Maslach C Schaufeli WB Leiter MP. Job burnout. Annu Rev Psychol 2001; 52: 397–422. 11148311 10.1146/annurev.psych.52.1.397

[3] Maslach C Jackson SE. Burnout in organizational settings. Appl Soc Psychol Ann 1984; 5: 133–153.

[4] Adriaenssens J de Gucht V Maes S. The impact of traumatic events on emergency room nurses: findings from a questionnaire survey. Int J Nurs Stud 2012; 49: 1411–1422. 22871313 10.1016/j.ijnurstu.2012.07.003

[5] Leape LL Shore MF Dienstag JL , et al. Perspective: a culture of respect, part 1: the nature and causes of disrespectful behavior by physicians. Acad Med 2012; 87: 845–852. 22622217 10.1097/ACM.0b013e318258338d

[6] Dubale BW Friedman LE Chemali Z , et al. Systematic review of burnout among healthcare providers in sub-Saharan Africa. BMC Public Health 2019; 19: 1247. 31510975 10.1186/s12889-019-7566-7 PMC6737653

[7] Yi Tay W Earnest A Yun Tan S , et al. Prevalence of burnout among nurses in a community hospital in Singapore: a cross-sectional study. Proc Singapore Healthcare 2014; 23(2): 93–99.

[8] Lasebikan VO Oyetunde MO. Burnout among nurses in a Nigerian General Hospital: prevalence and associated factors. ISRN Nurs 2012; 2012: 1–6. 10.5402/2012/402157 PMC3350958 22619733

[9] van der Doef M Mbazzi FB Verhoeven C . Job conditions, job satisfaction, somatic complaints and burnout among East African nurses. J Clin Nurs 2012; 21: 1763–1775. 22458703 10.1111/j.1365-2702.2011.03995.x

[10] Thorsen VC Tharp ALT Meguid T. High rates of burnout among maternal health staff at a referral hospital in Malawi: a cross-sectional study. BMC Nurs 2011; 10: 1–7. 21605379 10.1186/1472-6955-10-9 PMC3114002

[11] Chitura D Chitura M. Burnout syndrome in intensive care unit nurses in Zimbabwe. European Scientific Institute, https://eujournal.org/index.php/esj/article/view/4049 (2014, accessed 28 May 2018).

[12] Coetzee SK Klopper HC Ellis SM , et al. A tale of two systems-nurses practice environment, well being, perceived quality of care and patient safety in private and public hospitals in South Africa: a questionnaire survey. Int J Nurs Stud 2013; 50: 162–173. 23218020 10.1016/j.ijnurstu.2012.11.002

[13] Ledikwe JH Kleinman NJ Mpho M , et al. Associations between healthcare worker participation in workplace wellness activities and job satisfaction, occupational stress and burnout: a cross-sectional study in Botswana. BMJ Open 2018; 8: 1–7. 10.1136/bmjopen-2017-018492 PMC5857656 29549200

[14] Constable JF Russell DW. The effect of social support and the work environment upon burnout among nurses. J Human Stress 1986; 12: 20–26. 10.1080/0097840X.1986.9936762 3559184

[15] Kabir H Chowdhury SR Roy AK , et al. Association of workplace bullying and burnout with nurses’ suicidal ideation in Bangladesh. Sci Rep 2023; 13: 14641. 37669987 10.1038/s41598-023-41594-4 PMC10480219

[16] Gureje O Hollins S Botbol M , et al. Report of the WPA task force on brain drain. World Psychiatry 2009; 8: 115–118. 19516936 10.1002/j.2051-5545.2009.tb00225.x PMC2694032

[17] Cañadas-De la Fuente GA Vargas C San Luis C , et al. Risk factors and prevalence of burnout syndrome in the nursing profession. Int J Nurs Stud 2015; 52: 240–249. 25062805 10.1016/j.ijnurstu.2014.07.001

[18] Chowdhury SR Kabir H Akter N , et al. Impact of workplace bullying and burnout on job satisfaction among Bangladeshi nurses: a cross-sectional study. Heliyon 2023; 9: e13162. 10.1016/j.heliyon.2023.e13162 PMC9900271 36755612

[19] Alacacioglu A Yavuzsen T Dirioz M , et al. Burnout in nurses and physicians working at an oncology department. Psychooncology 2009; 18: 543–548. 18942658 10.1002/pon.1432

[20] Ndetei DM Pizzo M Maru H , et al. Burnout in staff working at the Mathari psychiatric hospital. Afr J Psychiatry (Johannesbg) 2008; 11: 199–203. 19588043 10.4314/ajpsy.v11i3.30269

[21] Schaufeli WB Enzmann D. The burnout companion to study and practice: a critical analysis. London, UK: Taylor & Francis, 1998.

[22] Tununu AF Martin P. Prevalence of burnout among nurses working at a psychiatric hospital in the Western Cape. Curationis 2020; 43: e1–e7. 10.4102/curationis.v43i1.2117 PMC7479374 32787430

[23] Tang Y Wang Y Zhou H , et al. The relationship between psychiatric nurses’ perceived organizational support and job burnout: mediating role of psychological capital. Front Psychol 2023; 14: 1099687. 36895741 10.3389/fpsyg.2023.1099687 PMC9989200

[24] Wang B Lu Q Sun F , et al. The relationship between sleep quality and psychological distress and job burnout among Chinese psychiatric nurses. Ind Health 2021; 59: 427–435. 34588380 10.2486/indhealth.2020-0249 PMC8655749

[25] Hasan AA Tumah H. The correlation between occupational stress, coping strategies, and the levels of psychological distress among nurses working in mental health hospital in Jordan. Perspect Psychiatr Care 2019; 55: 153–160. 29781526 10.1111/ppc.12292

[26] Zarea K Fereidooni-Moghadam M Baraz S , et al. Challenges encountered by nurses working in acute psychiatric wards: a qualitative study in Iran. Issues Ment Health Nurs 2018; 39: 244–250. 29064747 10.1080/01612840.2017.1377327

[27] Elsayed S Hasan AA Musleh M. Work stress, coping strategies and levels of depression among nurses working in mental health hospital in Port-Said city. Int J Cult Ment Health 2018; 11: 157–170.

[28] van der Colff JJ Rothmann S . Burnout of registered nurses in South Africa. J Nurs Manag 2014; 22: 630–642. 23405857 10.1111/j.1365-2834.2012.01467.x

[29] Spector PE. Measurement of human service staff satisfaction: development of the job satisfaction survey. Am J Community Psychol 1985; 13: 693. 4083275 10.1007/BF00929796

[30] van Saane N . Reliability and validity of instruments measuring job satisfaction—a systematic review. Occup Med (Chic Ill) 2003; 53: 191–200. 10.1093/occmed/kqg038 12724553

[31] O’Keefe DF Kelloway EK Francis R. Introducing the OCEAN.20: a 20-item five-factor personality measure based on the trait self-descriptive inventory. Milit Psychol 2012; 24: 433–460.

[32] Ghasemi A Zahediasl S. Normality tests for statistical analysis: a guide for non-statisticians. Int J Endocrinol Metab 2012; 10: 486. 23843808 10.5812/ijem.3505 PMC3693611

[33] Tabachnick BG Fidell LS. Using multivariate statistics. 7th ed. New York, NY: Pearson Education, 2013.

[34] Buitendach JH Moola MA. Coping occupational wellbeing and job satisfaction of nurses. J Psychol Afr 2011; 21: 43–52.

[35] Zarei E Ahmadi F Sial MS , et al. Prevalence of burnout among primary health care staff and its predictors: a study in Iran. Int J Environ Res Public Health 2019; 16: 2249. 31242691 10.3390/ijerph16122249 PMC6616853

[36] Engelbrecht MC Bester CL Van Den Berg H , et al. A study of predictors and levels of burnout: the case of professional nurses in primary health care facilities in the free state. South Afr J Econ 2008; 76: 15–27.

[37] Sahraian A Fazelzadeh A Mehdizadeh AR , et al. Burnout in hospital nurses: a comparison of internal, surgery, psychiatry and burns wards. Int Nurs Rev 2008; 55: 62–67. 18275537 10.1111/j.1466-7657.2007.00582.x

[38] Okwaraji FE Aguwa EN. Burnout and psychological distress among nurses in a Nigerian tertiary health institution. Afr Health Sci 2014; 14: 237–245. 26060486 10.4314/ahs.v14i1.37 PMC4449076

[39] Wu H Liu L Sun W , et al. Factors related to burnout among Chinese female hospital nurses: Cross-sectional survey in Liaoning province of China. J Nurs Manag 2014; 22: 621–629. 25041802 10.1111/jonm.12015

[40] Tekindal B Tekindal MA Pinar G , et al. Nurses’ burnout and unmet nursing care needs of patients’ relatives in a Turkish State Hospital. Int J Nurs Pract 2012; 18: 68–76. 22257333 10.1111/j.1440-172X.2011.01989.x

[41] Al-Turki H Al-Turki R Al-Dardas H , et al. Burnout syndrome among multinational nurses working in Saudi Arabia. Ann Afr Med 2010; 9: 226. 20935422 10.4103/1596-3519.70960

[42] Payne A Koen L Niehaus DJH , et al. Burnout and job satisfaction of nursing staff in a south African acute mental health setting. South Afr J Psychiatry 2020; 26: 1–6. 10.4102/sajpsychiatry.v26i0.1454 PMC7433218 32832126

[43] Lu H Barriball KL Zhang X , et al. Job satisfaction among hospital nurses revisited: a systematic review. Int J Nurs Stud 2012; 49: 1017–1038. 22189097 10.1016/j.ijnurstu.2011.11.009

[44] Kamal Bahrain NN Raihan Sakrani SN Maidin A. Communication barriers in work environment: understanding impact and challenges. Int J Acad Res Bus Soc Sci 2023; 13: 1489–1503. DOI: 10.6007/ijarbss/v13-i11/19498.

[45] Darban F Balouchi A Narouipou A , et al. Effect of communication skills training on the burnout of nurses: a cross-sectional study. J Clin Diagn Res 2016; 10: IC01–IC04. 10.7860/JCDR/2016/19312.7667 PMC4866130 27190832

[46] Gabriel KP Aguinis H. How to prevent and combat employee burnout and create healthier workplaces during crises and beyond. Bus Horiz 2022; 65: 183–192.

[47] Ang SY Dhaliwal SS Ayre TC , et al. Demographics and personality factors associated with burnout among nurses in a Singapore Tertiary Hospital. Biomed Res Int 2016; 2016: 6960184. 27478835 10.1155/2016/6960184 PMC4960324

[48] Molavynejad S Babazadeh M Bereihi F , et al. Relationship between personality traits and burnout in oncology nurses. J Fam Med Prim Care 2019; 8: 2898. 10.4103/jfmpc.jfmpc_423_19 PMC6820379 31681663

[49] Ecie MT Biderman MD Cunningham CJL , et al. Relationships among nursing burnout, the big five personality factors, and overall self-concept: The impact of assessing common method variance. J Soc Psychol 2013; 146(1): 31–50.

[50] Lee SY Ohtake F. Is being agreeable a key to success or failure in the labor market? J Jpn Int Econ 2018; 49: 8–27.

[51] Reizer A Harel T Ben-Shalom U. Helping others results in helping yourself: how well-being is shaped by agreeableness and perceived team cohesion. Behav Sci 2023; 13: 150. 36829379 10.3390/bs13020150 PMC9952276

