# Supplementary material for: Burnout and associated factors among nurses in psychiatric and general tertiary hospitals in Botswana: A cross-sectional study
Source: SAGE Open Med. 2024 Oct 9;12:20503121241272636. doi: 10.1177/20503121241272636 (PMC11462561; doi:10.1177/20503121241272636)
Supplement: sj-docx-1-smo-10.1177_20503121241272636 – Supplemental material for Burnout and associated factors among nurses in psychiatric and general tertiary hospitals in Botswana: A cross-sectional study [file sj-docx-1-smo-10.1177_20503121241272636.docx]

|  | **SOCIO DEMOGRAPHIC QUESTIONNAIRE**  **Topic: BURNOUT AMONG NURSES IN A REFERRAL PSYCHIATRIC HOSPITAL AND A REFFERAL MEDICAL HOSPITAL: PREVALENCE, RISK AND PROTECTIVE FACTORS**  **Serial Code ………..** | |
| --- | --- | --- |
| 1 | Date of completion of the survey | └─┴─┘ └─┴─┘ └─┴─┴─┴─┘  dd mm year |
| 2 | Hospital | ______________________________________ |
| 3 | How long have you been working in the current hospital? | - 1. Year   2-5 years  6-10 years  Above 10 years |
|  | **Demographic Information** |  |
| 4 | Sex | Male …………..  Female ………... |
| 5 | How old are you? | Years └─┴─┘ |
| 6 | What is your marital status? | Never married  Currently married  Divorced  Widowed  Cohabiting  Others |
| 7 | How many people live in your household?  Children | Number of people └─┴─┘  Number of children └─┴─┘ |
| 8 | What is the highest level of education you have completed? | Diploma  Bachelor’s degree  Post-graduate |
| 9 | Current designation | ___________________________ |
| 10 | What is your average income | Below 10,000  11,000-20,000  21,000-30,000  31,000-40,00  Above 40,000……………….  Not disclosed ………… |
| 11 | Which area are you currently based? (ICU, A&E, Oncology, theatre, OPD etc) | ___________________________ |
| 12 | If you have been in the current station for under three months, where were you previously based | __________________________ |
| 13 | Do you undertake any administrative duties? | Yes  No |
| 14 | What shift do you mostly do? | Day  Night  Both equally |

**MBI Human Services Survey, Christina Maslach – Susan E. Jackson**

The purpose of this survey is to find out how you feel about your job there are 22 statements of job-related feelings. Please read each statement carefully and decide if you ever feel this way *about your job.* If you have *never* had this feeling, write a "0" (zero) before the statement. If you have had this feeling, indicate *how often* you feel it by writing the number (from 1 to 6) that best describes how frequently you feel that way.

| **How Often** | **0** | **1** | **2** | **3** | **4** | **5** | **6** |
| --- | --- | --- | --- | --- | --- | --- | --- |
|  | Never | A few times a year or less | Once a month or less | A few times a month | Once a week | A few times a week | Every day |

| NO | ITEM | SCORE | | |
| --- | --- | --- | --- | --- |
|  |  | EE | DP | PA |
| 1 | I feel used up at the end of my workday |  |  |  |
| 2 | I feel I am at the end of my rope |  |  |  |
| 3 | I feel emotionally drained from my work |  |  |  |
| 4 | I feel very energetic |  |  |  |
| 5 | I have the feeling some recipients blame me for their problems |  |  |  |
| 6 | I feel frustrated by my job |  |  |  |
| 7 | I feel exhilarated after working closely with my recipients |  |  |  |
| 8 | I feel burned out from my job |  |  |  |
| 9 | I can easily create a relaxed atmosphere with my recipients |  |  |  |
| 10 | I feel I treat some recipients as if they were impersonal objects |  |  |  |
| 11 | I have become more callous towards people since I took the job |  |  |  |
| 12 | I feel I am positively influencing other people’s lives through my work |  |  |  |
| 13 | I feel I am working too much on my job |  |  |  |
| 14 | Working with people directly puts too much stress on me |  |  |  |
| 15 | I don’t really care what happens to some recipients |  |  |  |
| 16 | Working with people all day is really a strain for me |  |  |  |
| 17 | I deal very effectively with the problems of my recipients |  |  |  |
| 18 | I can easily understand how recipients feel about things |  |  |  |
| 19 | In my work, I deal with emotional problems very calmly |  |  |  |
| 20 | I feel tired when I get up in the morning and have to face another day on the job |  |  |  |
| 21 | I have accomplished many worthwhile things in this job |  |  |  |
| 22 | I worry that this job is worrying me emotionally |  |  |  |
| **TOTAL SCORE** | |  |  |  |

**The OCEAN.20^[[1]](#footnote-1)^**

**Occupational Personality Questionnaire^[[2]](#footnote-2)^**

This questionnaire asks about a variety of subjects such as your attitudes towards other people, what you like doing, and how you would feel in particular circumstances. The inventory takes about 5 minutes to complete, however there is no time limit.

**Please read the following instructions carefully.**

On the following page, you will find a number of adjectives and statements. Using the following rating scale, decide how well each of them describes you.

**1 - Extremely Uncharacteristic**

**2 - Quite Uncharacteristic**

**3 - Slightly Uncharacteristic**

**4 - Neither Characteristic Nor Uncharacteristic**

**5 - Slightly Characteristic**

**6 - Quite Characteristic**

**7 - Extremely Characteristic**

Here is an example: **‘Cautious’**

Decide if ‘cautious’ is characteristic of you. If you decide **‘cautious’** is ‘quite uncharacteristic’ of you, then you should select the number 2, which corresponds to the ‘quite uncharacteristic’ rating.

***Please reply to all adjectives and statements***. Give your first impression of how characteristic each adjective and statement is of you. Don’t spend too long on deciding what your answer should be. Answer all questions, even if you are not entirely sure of your answer.

**Answer honestly**. Please respond as honestly and accurately as you can.

| 1 | 2 | 3 | 4 | 5 | 6 | 7 |
| --- | --- | --- | --- | --- | --- | --- |
| Extremely Uncharacteristic | Quite Uncharacteristic | Slightly Uncharacteristic | Neither Characteristic Nor Uncharacteristic | Slightly Characteristic | Quite Characteristic | Extremely Characteristic |

| 1 | Silent | 1 | 2 | 3 | 4 | 5 | 6 | 7 |
| --- | --- | --- | --- | --- | --- | --- | --- | --- |
| 2 | Neat | 1 | 2 | 3 | 4 | 5 | 6 | 7 |
| 3 | Sympathetic | 1 | 2 | 3 | 4 | 5 | 6 | 7 |
| 4 | Organized | 1 | 2 | 3 | 4 | 5 | 6 | 7 |
| 5 | Withdrawn | 1 | 2 | 3 | 4 | 5 | 6 | 7 |
| 6 | Kind | 1 | 2 | 3 | 4 | 5 | 6 | 7 |
| 7 | Quiet | 1 | 2 | 3 | 4 | 5 | 6 | 7 |
| 8 | I have thought a lot about the origins of the universe | 1 | 2 | 3 | 4 | 5 | 6 | 7 |
| 9 | I like to keep all my belongings neat and organized | 1 | 2 | 3 | 4 | 5 | 6 | 7 |
| 10 | I often have headaches when things are not going well | 1 | 2 | 3 | 4 | 5 | 6 | 7 |
| 11 | I am always generous when it comes to helping others | 1 | 2 | 3 | 4 | 5 | 6 | 7 |
| 12 | Sometimes I get so upset, I feel sick to my stomach | 1 | 2 | 3 | 4 | 5 | 6 | 7 |
| 13 | I am highly interested in all fields of science | 1 | 2 | 3 | 4 | 5 | 6 | 7 |
| 14 | I like to have a place for everything and everything in its place | 1 | 2 | 3 | 4 | 5 | 6 | 7 |
| 15 | I am fascinated with the theory of evolution | 1 | 2 | 3 | 4 | 5 | 6 | 7 |
| 16 | When I am under great stress I often feel like I am about to break down | 1 | 2 | 3 | 4 | 5 | 6 | 7 |
| 17 | I always treat other people with kindness | 1 | 2 | 3 | 4 | 5 | 6 | 7 |
| 18 | My feelings are easily hurt | 1 | 2 | 3 | 4 | 5 | 6 | 7 |
| 19 | I am a very shy person | 1 | 2 | 3 | 4 | 5 | 6 | 7 |
| 20 | I would enjoy being a theoretical scientist | 1 | 2 | 3 | 4 | 5 | 6 | 7 |

|  | **JOB SATISFACTION SURVEY**  Paul E. Spector  Department of Psychology  University of South Florida  Copyright Paul E. Spector 1994, All rights reserved. |  |
| --- | --- | --- |
|  | PLEASE CIRCLE THE ONE NUMBER FOR EACH QUESTION THAT COMES CLOSEST TO REFLECTING YOUR OPINION  ABOUT IT. | Disagree very much  Disagree moderately  Disagree slightly  Agree slightly  Agree moderately  Agree very much |
| 1 | I feel I am being paid a fair amount for the work I do. | 1 2 3 4 5 6 |
| 2 | There is really too little chance for promotion on my job. | 1 2 3 4 5 6 |
| 3 | My supervisor is quite competent in doing his/her job. | 1 2 3 4 5 6 |
| 4 | I am not satisfied with the benefits I receive. | 1 2 3 4 5 6 |
| 5 | When I do a good job, I receive the recognition for it that I should receive. | 1 2 3 4 5 6 |
| 6 | Many of our rules and procedures make doing a good job difficult. | 1 2 3 4 5 6 |
| 7 | I like the people I work with. | 1 2 3 4 5 6 |
| 8 | I sometimes feel my job is meaningless. | 1 2 3 4 5 6 |
| 9 | Communications seem good within this organization. | 1 2 3 4 5 6 |
| 10 | Raises are too few and far between. | 1 2 3 4 5 6 |
| 11 | Those who do well on the job stand a fair chance of being promoted. | 1 2 3 4 5 6 |
| 12 | My supervisor is unfair to me. | 1 2 3 4 5 6 |
| 13 | The benefits we receive are as good as most other organizations offer. | 1 2 3 4 5 6 |
| 14 | I do not feel that the work I do is appreciated. | 1 2 3 4 5 6 |
| 15 | My efforts to do a good job are seldom blocked by red tape. | 1 2 3 4 5 6 |
| 16 | I find I have to work harder at my job because of the incompetence of people I work with. | 1 2 3 4 5 6 |
| 17 | I like doing the things I do at work. | 1 2 3 4 5 6 |
| 18 | The goals of this organization are not clear to me. | 1 2 3 4 5 6 |

|  | PLEASE CIRCLE THE ONE NUMBER FOR EACH QUESTION THAT COMES CLOSEST TO REFLECTING YOUR OPINION  ABOUT IT.  Copyright Paul E. Spector 1994, All rights reserved. | Disagree very mcuh  Disagree moderately  Disagree slightly  Agree slightly  Agree moderately  Agree very much |
| --- | --- | --- |
| 19 | I feel unappreciated by the organization when I think about what they pay me. | 1 2 3 4 5 6 |
| 20 | People get ahead as fast here as they do in other places. | 1 2 3 4 5 6 |
| 21 | My supervisor shows too little interest in the feelings of subordinates. | 1 2 3 4 5 6 |
| 22 | The benefit package we have is equitable. | 1 2 3 4 5 6 |
| 23 | There are few rewards for those who work here. | 1 2 3 4 5 6 |
| 24 | I have too much to do at work. | 1 2 3 4 5 6 |
| 25 | I enjoy my coworkers. | 1 2 3 4 5 6 |
| 26 | I often feel that I do not know what is going on with the organization. | 1 2 3 4 5 6 |
| 27 | I feel a sense of pride in doing my job. | 1 2 3 4 5 6 |
| 28 | I feel satisfied with my chances for salary increases. | 1 2 3 4 5 6 |
| 29 | There are benefits we do not have which we should have. | 1 2 3 4 5 6 |
| 30 | I like my supervisor. | 1 2 3 4 5 6 |
| 31 | I have too much paperwork. | 1 2 3 4 5 6 |
| 32 | I don't feel my efforts are rewarded the way they should be. | 1 2 3 4 5 6 |
| 33 | I am satisfied with my chances for promotion. | 1 2 3 4 5 6 |
| 34 | There is too much bickering and fighting at work. | 1 2 3 4 5 6 |
| 35 | My job is enjoyable. | 1 2 3 4 5 6 |
| 36 | Work assignments are not fully explained. | 1 2 3 4 5 6 |

1. O’Keefe, D.F., Kelloway, E. K., Francis, R. (2012). Introducing the OCEAN.20: A 20-item Five-Factor personality measure based on the Trait Self-Descriptive Inventory. Military Psychology, 24(5), 2012, 43-460. [↑](#footnote-ref-1)
2. This questionnaire is based on the Trait Self-Descriptive Inventory-Personality Inventory, which is the property of the Director General Military Personnel Research and Analysis, Canadian Department of National Defence. Questions and/or comments can be addressed to Damian.O’Keefe@forces.gc.ca [↑](#footnote-ref-2)
