# Supplementary material for: Burnout and associated factors among nurses in psychiatric and general tertiary hospitals in Botswana: A cross-sectional study
Source: SAGE Open Med. 2024 Oct 9;12:20503121241272636. doi: 10.1177/20503121241272636 (PMC11462561; doi:10.1177/20503121241272636)
Supplement: sj-docx-2-smo-10.1177_20503121241272636 – Supplemental material for Burnout and associated factors among nurses in psychiatric and general tertiary hospitals in Botswana: A cross-sectional study [file sj-docx-2-smo-10.1177_20503121241272636.docx]

**Supplementary table 1**

| **Burnout subscales** | **Independent variables** | **N** | **Mean** | **Std. Deviation** | **t** | **p-value** |
| --- | --- | --- | --- | --- | --- | --- |
|  | **Current position** |  |  |  |  |  |
| **Emotional exhaustion** | Junior | 63 | 27.6 | 14.4 | 1.22 | 0.225 |
|  | Middle to senior | 162 | 25.1 | 13.5 |  |  |
| **Depersonalization** | Junior | 63 | 7.8 | 6.3 | 0.85 | 0.397 |
|  | Middle to senior | 162 | 7.1 | 5.4 |  |  |
| **Personal achievement** | Junior | 63 | 35.7 | 8.5 | 2.13 | **0.036** |
|  | Middle to senior | 162 | 33.0 | 7.6 |  |  |
|  | **Marital status** |  |  |  |  |  |
| **Emotional exhaustion** | Single | 149 | 26.0 | 14.0 | 0.06 | 0.956 |
|  | Married | 98 | 25.9 | 14.1 |  |  |
| **Depersonalization** | Single | 149 | 7.4 | 5.8 | -0.11 | 0.913 |
|  | Married | 98 | 7.4 | 5.4 |  |  |
| **Emotional exhaustion** | Single | 149 | 34.3 | 8.1 | 1.27 | 0.205 |
|  | Married | 98 | 32.9 | 8.1 |  |  |
|  | **Gender** |  |  |  |  |  |
| **Emotional exhaustion** | Male | 88 | 25.3 | 14.5 | -0.65 | 0.515 |
|  | female | 159 | 26.5 | 13.8 |  |  |
| **Depersonalization** | Male | 88 | 7.4 | 5.5 | 0.11 | 0.910 |
|  | female | 159 | 7.4 | 5.6 |  |  |
| **Personal achievement** | Male | 88 | 32.9 | 9.0 | -1.29 | 0.197 |
|  | female | 159 | 34.3 | 7.5 |  |  |
|  | **Hospital** |  |  |  |  |  |
| **Emotional exhaustion** | Sbrana | 98 | 21.8 | 12.6 | -3.934 | **<0.01** |
|  | Marina | 147 | 28.8 | 14.3 |  |  |
| **Depersonalization** | Sbrana | 98 | 6.51 | 4.8 | -1.93 | **0.045** |
|  | Marina | 147 | 7.90 | 5.9 |  |  |
| **Personal achievement** | Sbrana | 98 | 33.1 | 7.8 | -1.00 | 0.316 |
|  | Marina | 147 | 34.1 | 8.3 |  |  |
