# Supplementary material for: Burnout and associated factors among nurses in psychiatric and general tertiary hospitals in Botswana: A cross-sectional study
Source: SAGE Open Med. 2024 Oct 9;12:20503121241272636. doi: 10.1177/20503121241272636 (PMC11462561; doi:10.1177/20503121241272636)
Supplement: sj-docx-3-smo-10.1177_20503121241272636 – Supplemental material for Burnout and associated factors among nurses in psychiatric and general tertiary hospitals in Botswana: A cross-sectional study [file sj-docx-3-smo-10.1177_20503121241272636.docx]

**Supplementary table 2**

| **Variables** | **1** | **2** | **3** | **4** | **5** | **6** | **7** | **8** | **9** | **10** | **11** | **12** | **13** | **14** | **15** | **16** | **17** | **18** | **19** | **20** | **21** |
| --- | --- | --- | --- | --- | --- | --- | --- | --- | --- | --- | --- | --- | --- | --- | --- | --- | --- | --- | --- | --- | --- |
| **1. Emotional exhaustion** | 1 | .59^**^ | .021 | -.032 | -.123 | -.021 | .089 | .203^**^ | .132^*^ | .010 | .113 | .301^**^ | .135^*^ | .066 | .143^*^ | .106 | .189^**^ | .360^**^ | .226^**^ | -.026 | 0.26^**^ |
| **2.Depersonalization** |  | 1 | -.050 | -.045 | .019 | .000 | .095 | .145^*^ | .080 | .003 | .033 | .241^**^ | .150^*^ | .055 | .202^**^ | .092 | .147^*^ | .255^**^ | .187^**^ | -.041 | 0.24^**^ |
| **3.Personal achievement** |  |  | 1 | -.036 | -.187^**^ | -.106 | -.139^*^ | .153^*^ | .265^**^ | -.043 | .329^**^ | .005 | -.112 | -.058 | .011 | -.023 | -.014 | .006 | .091 | .263^**^ | -.05 |
| **4. AGE** |  |  |  | 1 | .186^**^ | .354^**^ | .425^**^ | -.166^*^ | -.086 | -.031 | -.031 | -.026 | -.116 | -.209^**^ | -.065 | .004 | -.065 | -.078 | -.057 | -.088 | .01 |
| **5. No in the household** |  |  |  |  | 1 | .485^**^ | .051 | -.132^*^ | -.086 | -.047 | -.026 | .012 | -.101 | -.167^**^ | -.008 | -.093 | -.114 | -.038 | -.057 | -.135^*^ | -.05 |
| **6. No of children** |  |  |  |  |  | 1 | .276^**^ | -.109 | -.155^*^ | -.053 | -.183^**^ | .102 | -.050 | -.059 | -.036 | -.013 | -.019 | -.061 | -.027 | -.126 | .05 |
| **7.Time at current hospital** |  |  |  |  |  |  | 1 | -.057 | .017 | -.016 | .005 | .041 | -.090 | -.018 | .071 | .075 | .045 | .114 | .032 | -.092 | .02 |
| **8. Openness** |  |  |  |  |  |  |  | 1 | .326^**^ | .102 | .155^*^ | .280^**^ | .006 | .015 | -.024 | -.080 | .080 | .056 | .107 | .187^**^ | .074 |
| **9. Conscientiousness** |  |  |  |  |  |  |  |  | 1 | .085 | .674^**^ | .105 | .022 | -.018 | .008 | -.054 | .032 | .036 | .007 | .125 | -.047 |
| **10. Extraversion** |  |  |  |  |  |  |  |  |  | 1 | .109 | .265^**^ | -.039 | -.046 | -.067 | -.062 | .055 | -.118 | -.005 | -.089 | -.103 |
| **11. Agreeableness** |  |  |  |  |  |  |  |  |  |  | 1 | .078 | -.113 | -.096 | -.055 | -.063 | -.014 | .046 | .005 | .125 | -.060 |
| **12. Neuroticism** |  |  |  |  |  |  |  |  |  |  |  | 1 | .011 | .082 | .132^*^ | .117 | .107 | .098 | .137^*^ | .044 | .133^*^ |
| **13. Pay** |  |  |  |  |  |  |  |  |  |  |  |  | 1 | .255^**^ | .310^**^ | .350^**^ | .305^**^ | .349^**^ | .253^**^ | .322^**^ | .242^**^ |
| **14. Promotion** |  |  |  |  |  |  |  |  |  |  |  |  |  | 1 | .210^**^ | .397^**^ | .432^**^ | .196^**^ | .162^*^ | .188^**^ | .170^**^ |
| **15. Supervision** |  |  |  |  |  |  |  |  |  |  |  |  |  |  | 1 | .330^**^ | .328^**^ | .366^**^ | .409^**^ | .272^**^ | .342^**^ |
| **16. Fringe benefit** |  |  |  |  |  |  |  |  |  |  |  |  |  |  |  | 1 | .325^**^ | .328^**^ | .254^**^ | .254^**^ | .302^**^ |
| **17. Contingency reward** |  |  |  |  |  |  |  |  |  |  |  |  |  |  |  |  | 1 | .395^**^ | .362^**^ | .318^**^ | .300^**^ |
| **18. Operating condition** |  |  |  |  |  |  |  |  |  |  |  |  |  |  |  |  |  | 1 | .433^**^ | .232^**^ | .309^**^ |
| **19. Coworker** |  |  |  |  |  |  |  |  |  |  |  |  |  |  |  |  |  |  | 1 | .356^**^ | .313^**^ |
| **20. Nature of work** |  |  |  |  |  |  |  |  |  |  |  |  |  |  |  |  |  |  |  | 1 | .131^*^ |
| **21. Communication** |  |  |  |  |  |  |  |  |  |  |  |  |  |  |  |  |  |  |  | . | 1 |

Correlation is significant at the 0.01 level (2-tailed).

Correlation is significant at the 0.05 level (2-tailed).
